# Correlation of Echocardiographic Right Ventricular Functional Parameters with CMR-Derived Right Ventricular Ejection Fraction in Pulmonary Hypertension: A Systematic Review and Meta-Analysis

**DOI:** 10.3390/diagnostics16182899

**Published:** 2026-09-09

**Authors:** Faizan Ahmed, Ayesha Zulfiqar, Ramsha Ali, Syeda Simrah Shah, Saba Aliha, Muhammad Saad Khan, Sheraz Qasim, Maria Campwala, Maliha Khalid, Adiesh Sood, Umair Iqbal, Haris Bin Tahir, Nazila Dalir, Taha Alam, Ali Abbas Khaleel, Anand Reddy Maligireddy, Ritu Yadav, Amro Taha, Ali Ghani, Fawaz Alenezi

**Affiliations:** 1 Department of Internal Medicine, Jersey Shore University Medical Center, Hackensack Meridian Health, Neptune, NJ 07753, USA; 2 Department of Medicine, Dow University of Health Sciences, Karachi 74200, Pakistan; ayeshazulfiqar1725@gmail.com (A.Z.); syedaali969@gmail.com (S.S.S.); mariakhalid909@gmail.com (M.C.); 3 Department of Internal Medicine, Peoples University of Medical and Health Sciences, Nawabshah 67480, Pakistan; aliramsha235@gmail.com; 4 Department of Medicine, Multan Medical and Dental College, Multan 60000, Pakistan; sabaaliha23@icloud.com; 5 Department of Internal Medicine, Jinnah Sindh Medical University, Karachi 75510, Pakistan; mohammadsaadkhan000@gmail.com; 6 Department of Medicine, Ameer-ud-Din Medical College, Post Graduate Medical Institute, Lahore 54000, Pakistan; sherazqasim08@gmail.com; 7 Department of Medicine, Jinnah Sindh Medical University, Karachi 75510, Pakistan; malihakhalid2002@gmail.com; 8 Department of Medicine, Adesh Institute of Medical Sciences and Research, Bathinda 151101, Punjab, India; adishsood1101@gmail.com; 9 Department of Medicine, CMH Multan Institute of Medical Sciences, Multan 60000, Pakistan; umairiqbalhiraj@gmail.com; 10 Department of Medicine, Lahore General Hospital, Lahore 54700, Pakistan; harristahirchh@gmail.com; 11 School of Medicine, St. George’s University, St. George’s, Grenada; 12 Department of Internal Medicine, Ameer-ud-Din Medical College, Post Graduate Medical Institute, Lahore 54000, Pakistan; tahaaalam9@gmail.com; 13 Department of Medicine, College of Medicine, Al-Mustansiriyah University, Baghdad 10001, Iraq; dr.ali.abbas.k@gmail.com; 14 Department of Cardiovascular Medicine, The University of Kansas Medical Center, Kansas City, KS 66160, USA; amaligireddy@kumc.edu; 15 Department of Internal Medicine, Suny Upstate University, Syracuse, NY 13210, USA; rituyadav122@gmail.com; 16 Division of Cardiology, West Virginia University, Morgantown, WV 26506, USA; taha61591@gmail.com; 17 Division of Structural Cardiology, University of Arizona, Tucson, AZ 85721, USA; alighani152@gmail.com; 18 Division of Cardiology, Duke University School of Medicine, Durham, NC 27710, USA

**Keywords:** pulmonary hypertension chest imaging, diagnostic accuracy, meta-analysis, echocardiography, cardiac magnetic resonance (CMR)

## Abstract

**Background:** Pulmonary hypertension (PH) is associated with increased pulmonary arterial pressure and impairment of right ventricular (RV) function. Reliable characterization of RV performance is important for prognosis, risk assessment, and therapeutic planning. Cardiac magnetic resonance (CMR) is considered the reference technique for quantifying RV volumes and function, but its expense and limited availability can restrict routine implementation. Echocardiography is more widely accessible; however, the extent to which individual echocardiographic indices correspond with CMR-derived RV ejection fraction (RVEF) remains incompletely defined. **Objective:** Our objective was to determine the correlation of RV fractional area change (RV-FAC), RV free-wall longitudinal strain (RV-FWLS), and RV global longitudinal strain (RV-GLS) with CMR-derived RVEF in individuals with PH. **Methods:** PubMed, Embase, ClinicalTrials.gov, Scopus, and the Cochrane Library were searched, yielding 404 records; six studies fulfilled the eligibility criteria. Studies were eligible when they enrolled patients with PH and provided extractable correlations between at least one prespecified echocardiographic measure and CMR-derived RVEF. Correlation coefficients were converted with Fisher’s z transformation, combined using common-effect and random-effects approaches, and then transformed back to correlation coefficients. Pooled mean RV-FAC was evaluated as a separate outcome. Between-study heterogeneity was quantified with I^2^ and τ^2^, with random-effects estimates emphasized for interpretation. **Results:** Each evaluated echocardiographic measure was positively correlated with CMR-derived RVEF. RV-FWLS had the largest pooled correlation and the greatest consistency (r = 0.79, 95% CI: 0.73–0.83; I^2^ = 0.0%). The pooled correlation for RV-GLS was 0.75 (95% CI: 0.60–0.85; I^2^ = 80.3%), while that for RV-FAC was 0.72 (95% CI: 0.61–0.80; I^2^ = 61.8%). The random-effects pooled mean RV-FAC was 31.10% (95% CI: 26.77–35.43), with substantial heterogeneity (I^2^ = 90.9%). **Conclusions:** RV-FWLS showed the highest numerical and most consistent association with CMR-derived RVEF, suggesting that it may serve as a useful non-invasive adjunct for RV assessment in PH, especially where CMR is not readily available. Larger studies using harmonized acquisition and analysis methods are required for confirmation.

## 1. Introduction

Pulmonary hypertension (PH) is defined by elevated pulmonary arterial pressure and is frequently accompanied by right ventricular (RV) dysfunction. Impaired RV function is associated with disease progression and poorer outcomes [1], including reduced systolic performance, diminished cardiac output, hospitalization, and mortality. Right ventricular diastolic dysfunction (RVDD) has also been identified as an important prognostic feature in pulmonary arterial hypertension (PAH) [2]. Because RV performance strongly influences symptoms and prognosis, assessment should be accurate, reproducible, and suitable for serial evaluation. The interaction between pulmonary vascular load and RV contractile adaptation, termed right ventricular–pulmonary arterial (RV–PA) coupling, is closely linked to the hemodynamic and functional effects of PH [1].

The RV is particularly challenging to quantify because of its irregular geometry and thin myocardial wall, which is approximately 3–4 mm thick [3]. Echocardiographic approaches have therefore expanded beyond conventional measurements to include three-dimensional imaging and speckle-tracking techniques [4]. Echocardiography remains the principal modality for RV assessment because it is relatively accessible, inexpensive, and convenient [5]. Commonly used indices include tricuspid annular plane systolic excursion (TAPSE), RV fractional area change (RV-FAC), RV free-wall longitudinal strain (RV-FWLS), and RV global longitudinal strain (RV-GLS). Nevertheless, evidence regarding how consistently these indices correspond with CMR-based measures remains variable.

Several factors can compromise echocardiographic RV assessment, including the chamber’s complex morphology, suboptimal image quality, operator dependence, and challenges in identifying the true endocardial boundary [6]. CMR offers more detailed structural characterization, permits clearer visualization of the RV myocardial contour, and allows direct quantification of ventricular volumes [6]. Its reproducibility and comprehensive assessment of cardiac structure and function have led to its use as the reference imaging modality for RV evaluation [7]. However, CMR may be impractical for routine or repeated assessment because of cost, limited availability, lengthy examinations, and patient-specific contraindications such as claustrophobia or certain implanted devices.

Although individual investigations have examined relationships between RV-FAC, RV-FWLS, RV-GLS, and CMR-derived RVEF, the magnitude and consistency of these associations across studies remain unclear. We therefore performed this systematic review and meta-analysis to evaluate and descriptively compare the correlations of these three echocardiographic measures with CMR-derived RVEF in patients with PH.

## 2. Methods

This systematic review and meta-analysis followed the Cochrane Handbook guidance and was reported in accordance with the Preferred Reporting Items for Systematic Reviews and Meta-Analyses (PRISMA) statement [8]. The review protocol was prospectively registered in PROSPERO (CRD420261365765).

### 2.1. Data Searches and Sources

We searched PubMed, Embase, ClinicalTrials.gov, Scopus, and the Cochrane Library from database inception through March 2025. The search was designed to identify studies examining associations between echocardiographic RV measurements and CMR-derived RVEF in PH. Controlled vocabulary and free-text terms were combined, including MeSH concepts for ‘Pulmonary Hypertension,’ ‘Echocardiography,’ ‘Magnetic Resonance Imaging,’ and ‘Ventricular Function, Right,’ together with terms describing PAH, chronic thromboembolic PH, RV function, speckle tracking, longitudinal strain, RV-FWLS, RV-GLS, RV-FAC, CMR, and RVEF. AND/OR operators were applied to construct database-specific search strategies.

### 2.2. Eligibility Criteria

We considered observational and comparative studies that examined associations between echocardiographic measures of RV function and CMR-derived RVEF in patients with PH. The prespecified echocardiographic measures were RV-FAC, RV-FWLS, and RV-GLS. Studies were excluded when PH was not represented, none of the prespecified echocardiographic parameters were assessed, CMR-derived RVEF was unavailable, or a usable correlation coefficient could not be obtained. Animal research, reviews, editorials, case reports, and conference abstracts lacking adequate quantitative information were also excluded.

### 2.3. Study Selection and Data Extraction

All retrieved records were imported into Rayyan for duplicate removal and screening. Two investigators independently reviewed titles and abstracts, followed by full-text assessment of potentially eligible reports against the predefined criteria. Disagreements concerning eligibility were resolved by discussion with a third investigator. Two reviewers independently entered relevant study information into a prespecified Excel data sheet; discrepancies were addressed through consensus or third-author adjudication.

### 2.4. Echocardiography

Included studies were required to report an association between CMR-derived RVEF and at least one of RV-FAC, RV-FWLS, or RV-GLS. Correlation coefficients were converted to Fisher’s z values for meta-analysis. After pooling, estimates and their confidence intervals were transformed back to the correlation scale to facilitate clinical interpretation.

### 2.5. Cardiac MRI Parameters

CMR-derived RVEF served as the reference indicator of RV systolic function. In the included studies, RV end-diastolic and end-systolic volumes were derived from cine CMR using protocols defined by each study, with RVEF calculated from those measurements. Where reported, we recorded CMR field strength, acquisition procedures, analysis software, contouring methodology, and the interval separating echocardiography from CMR, as differences in examination timing could contribute to variation in measured RV function.

### 2.6. Quality Assessment

Two investigators independently assessed methodological quality, compared their ratings, and resolved disagreements by consensus; a third reviewer was involved when agreement could not be reached. Observational studies were evaluated with the appropriate Newcastle–Ottawa Scale (NOS), which assigns up to nine stars across Selection (4 stars), Comparability (2 stars), and Outcome/exposure assessment (3 stars).

### 2.7. Statistical Analysis

Meta-analyses were performed in R version 4.5.2. Correlation coefficients were synthesized under both common-effect and random-effects models, with the random-effects approach emphasized because differences in clinical characteristics and methodology were anticipated. Mean values and correlation coefficients were treated as separate outcomes and were neither combined nor interpreted as equivalent measures. Heterogeneity was evaluated using I^2^ and τ^2^; I^2^ values above 50% were considered substantial. Fisher’s z transformation was applied before pooling correlation coefficients, followed by back-transformation for reporting. Because only a small number of studies contributed to each analysis, funnel-plot asymmetry and Egger’s test were not considered sufficiently reliable for assessment of publication bias.

## 3. Results

**Figure 1:**
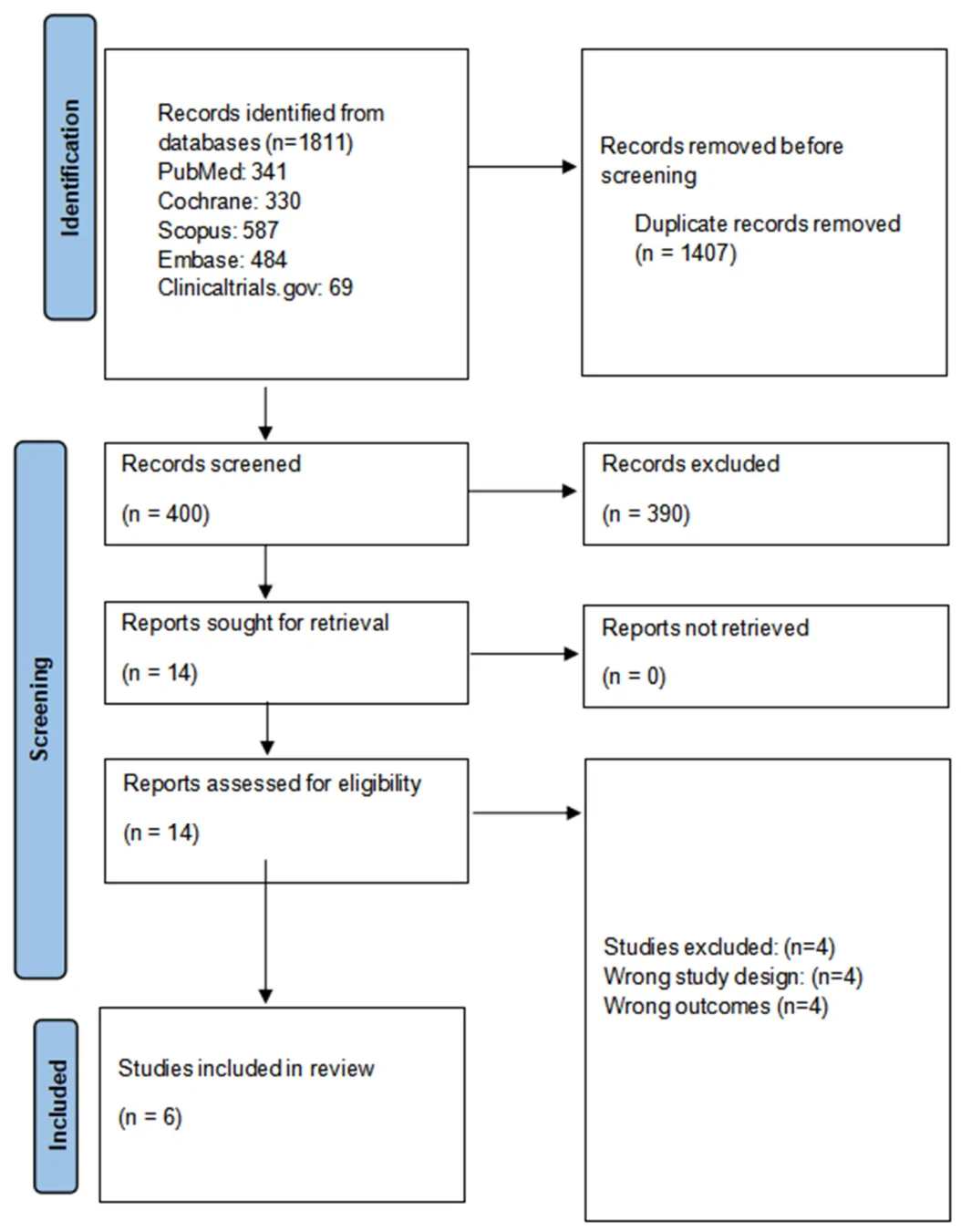
PRISMA flow diagram of the included studies.

Figure 1 summarizes the study-selection process. The identification stage yielded 1811 records: PubMed (341), Cochrane Library (330), Scopus (587), Embase (484), and ClinicalTrials.gov (69). Removal of 1407 duplicates left 404 unique reports for retrieval, all of which were available. Fourteen reports proceeded to eligibility assessment, and eight were excluded because their designs or outcomes did not meet the prespecified criteria. Six studies [5,9,10,11,12,13] were ultimately included in the systematic review and meta-analysis.

The PRISMA flowchart (Figure 1) illustrates the study selection process for this systematic review, beginning with the identification phase, where a total of 1811 records were sourced from databases (PubMed = 341, Cochrane = 330, Scopus = 587, Embase = 484) and registers (ClinicalTrials.gov = 69). After removing 1407 duplicate records, the remaining 404 reports were sought for retrieval, with none missing. In the eligibility assessment phase, 14 reports were evaluated, leading to the exclusion of 8 studies because of ineligible study designs or outcomes. Ultimately, 6 studies [5,9,10,11,12,13] met the inclusion criteria and were incorporated into the final review. This structured approach ensured a transparent and rigorous selection of relevant studies for systematic analysis.

### 3.1. Study and Baseline Characteristics

The six included studies were conducted in multiple countries and collectively enrolled 402 participants, with individual sample sizes ranging from 30 to 121. Mean participant age ranged from 30 ± 10 years in the prospective observational study by Yang et al. to 62 ± 15 years in the comparative cohort reported by Evaldsson et al. [9]. Sex distribution varied between cohorts; for example, women represented 80% of the participants in the prospective Chinese study by Yang et al. The study populations encompassed several forms of PH, including idiopathic PAH, connective tissue disease-associated PAH, chronic thromboembolic PH, and other etiologies. Additional baseline and study-level information is provided in Table 1 and Tables S1–S3. The interval between echocardiography and CMR also differed among studies, generally ranging from less than 3 h to 1 week, although one cohort reported that 90% of examinations occurred within 3 h and the remainder within 3 weeks. Detailed timing information is provided in Supplementary Table S5.

### 3.2. Quality Assessment

All six studies underwent quality appraisal with the Newcastle–Ottawa Scale (NOS). The assessment considered domains including representativeness of the study population, comparability and control of confounding, and outcome assessment. Scores of 7–9 stars were classified as high quality, 4–6 as moderate quality, and 0–3 as low quality. Each included study achieved a rating within the good-quality range, and none was classified as fair or poor. The complete assessments are presented in Table S4.

### 3.3. Echocardiographic Parameters

Because the pooled outcomes showed varying degrees of between-study heterogeneity, random-effects results were given primary emphasis. Pooled means and correlation coefficients describe different statistical quantities and were therefore presented and interpreted independently. The principal pooled estimates are summarized in Table 2.

**Figure 2:**
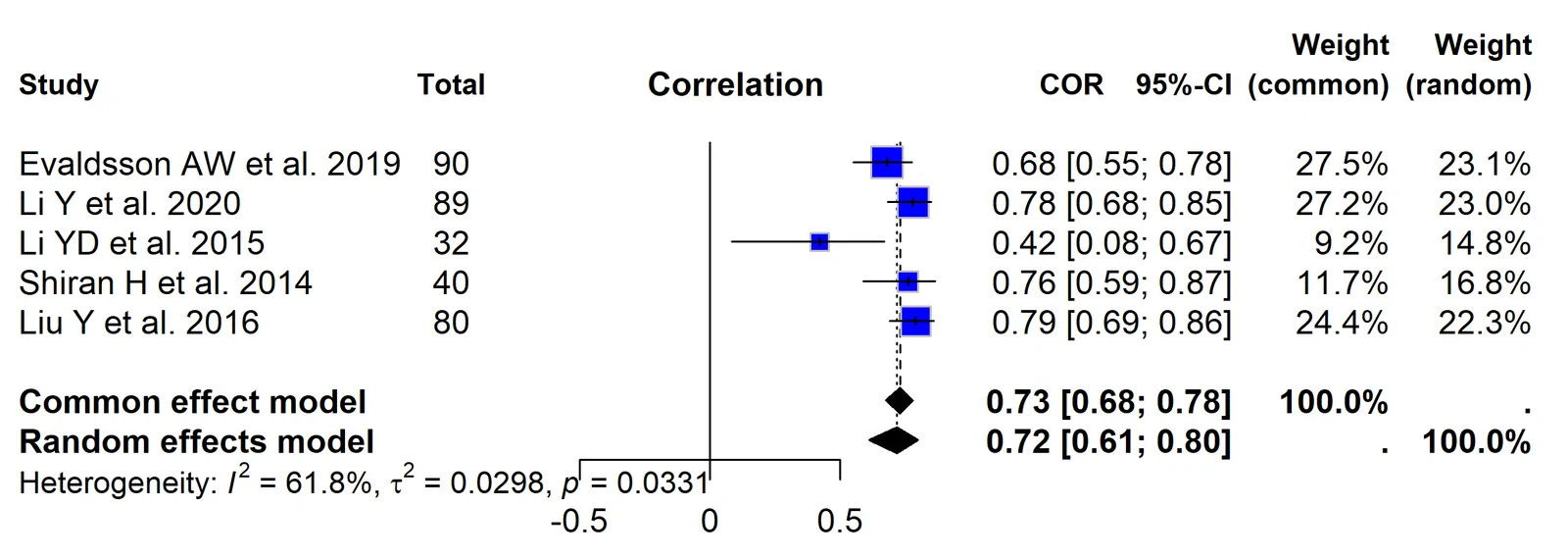
Forest plot of the right ventricular fractional area change correlation coefficient [5,9,10,11,13].

Eight RV-FAC mean estimates from five studies contributed 376 observations to the analysis. The random-effects pooled mean was 31.10% (95% CI: 26.77–35.43), with substantial heterogeneity (I^2^ = 90.9%, τ^2^ = 36.3947, and *p* < 0.0001). Across individual estimates, mean RV-FAC values ranged from 21.00% to 44.00%. Under the common-effect model, the pooled mean was 30.54% (95% CI: 29.58–31.49). Given the marked between-study variability, the random-effects estimate was used for primary interpretation (Figure 2).

RV-FAC mean values were reported in eight estimates from five studies, representing 376 observations in the forest plot. The random-effects pooled mean RV-FAC was 31.10% (95% CI: 26.77–35.43), with substantial heterogeneity (I^2^ = 90.9%, τ^2^ = 36.3947, and *p* < 0.0001). Individual mean values ranged from 21.00% to 44.00%. The common-effect model produced a pooled mean of 30.54% (95% CI: 29.58–31.49). Given the substantial between-study variability, the random-effects estimate was prioritized for interpretation (Figure 2).

**Figure 3:**
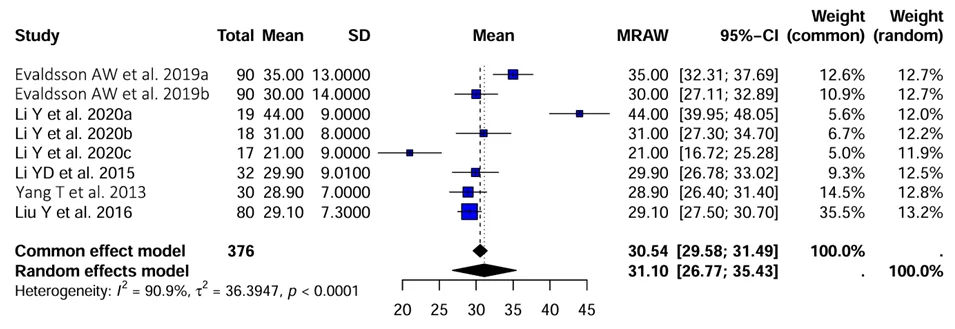
Forest plot of the mean right ventricular fractional area change [5,9,10,12,13].

Five studies comprising 331 participants reported the association between RV-FAC and CMR-derived RVEF. The random-effects synthesis showed a significant positive correlation (r = 0.72, 95% CI: 0.61–0.80). Heterogeneity was moderate to substantial (I^2^ = 61.8%, τ^2^ = 0.0298, and *p* = 0.0331), and individual study coefficients ranged from 0.42 to 0.79. The common-effect analysis yielded r = 0.73 (95% CI: 0.68–0.78); nevertheless, the random-effects estimate was retained as the primary result because of the observed between-study variability (Figure 3).

**Figure 4:**
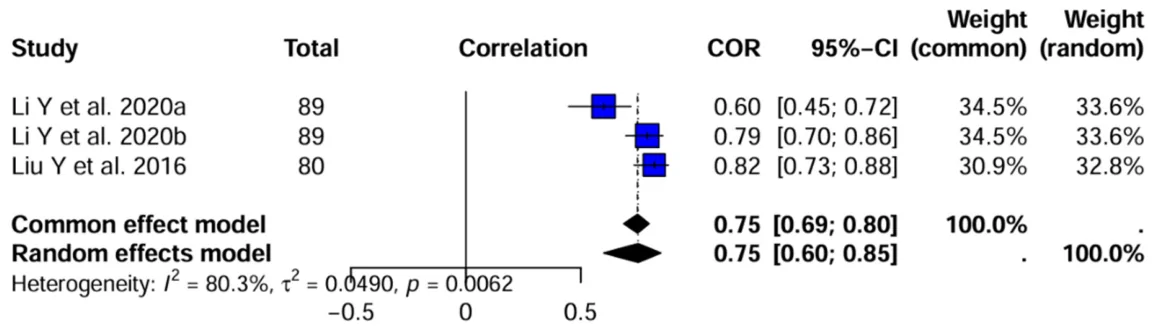
Forest plot of the RV-GLS correlation coefficient [10,13].

Three correlation estimates derived from two studies (sample sizes 89 and 80) were available for RV-GLS. The random-effects model produced a pooled correlation of 0.75 (95% CI: 0.60–0.85), with substantial heterogeneity (I^2^ = 80.3%, τ^2^ = 0.0490, and *p* = 0.0062). Individual coefficients ranged from 0.60 to 0.82, and all three estimates indicated statistically significant positive associations. The common-effect model produced the same pooled point estimate, r = 0.75 (95% CI: 0.69–0.80) (Figure 4).

RV-GLS demonstrated a significant positive correlation with CMR-derived RVEF in patients with PH. The analysis included three correlation estimates from two studies, involving study samples of 89 and 80 participants. The random-effects model yielded a pooled correlation coefficient of 0.75 (95% CI: 0.60–0.85), with substantial heterogeneity (I^2^ = 80.3%, τ^2^ = 0.0490, and *p* = 0.0062). Although the pooled estimate indicates a positive correlation, the wider confidence interval highlights the uncertainty introduced by inconsistency between the estimates. Individual correlation coefficients ranged from 0.60 to 0.82, and all three estimates showed statistically significant positive correlations. The common-effect model also yielded a pooled correlation coefficient of 0.75 (95% CI: 0.69–0.80) (Figure 4).

The RV-FWLS analysis incorporated three studies with 259 participants. A significant positive relationship with CMR-derived RVEF was observed, with a random-effects pooled correlation of 0.79 (95% CI: 0.73–0.83). No measurable between-study heterogeneity was identified (I^2^ = 0.0%, τ^2^ = 0, and *p* = 0.9809), indicating highly concordant estimates across the available studies.

**Figure 5:**
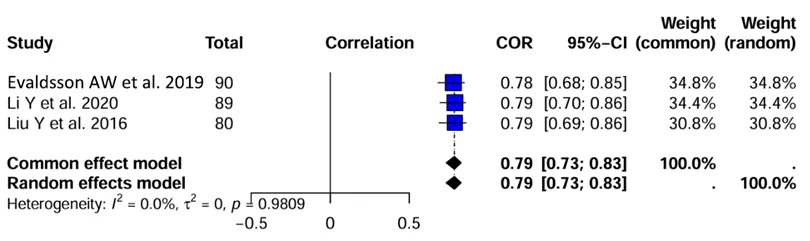
Forest plot of the RV-FWLS correlation coefficient [9,10,13].

The common-effect model produced the same pooled estimate, r = 0.79 (95% CI: 0.73–0.83). Individual study coefficients were statistically significant and ranged from 0.78 to 0.79, further indicating a strong and consistent association between RV-FWLS and CMR-derived RVEF (Figure 5).

**Figure 6:**
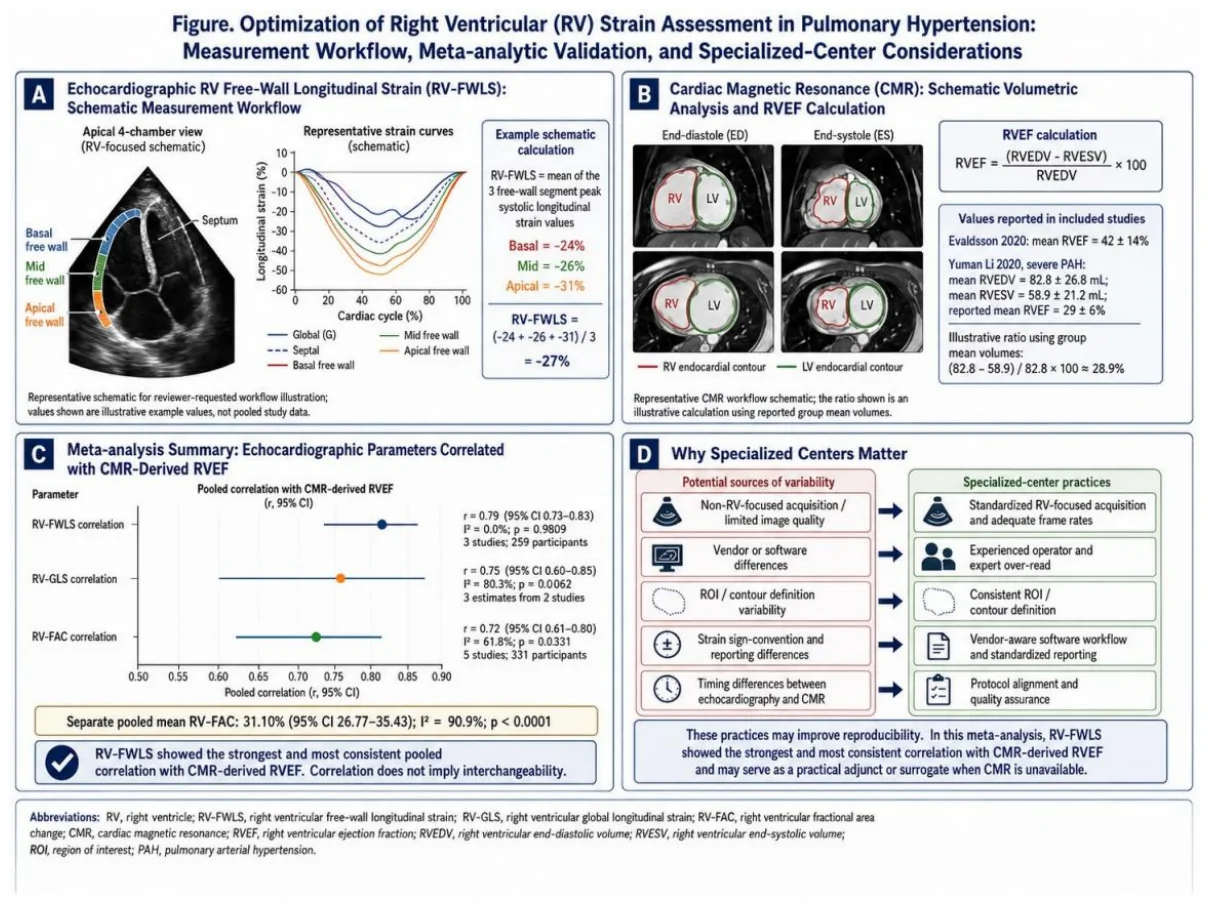
Integrated illustration of echocardiographic and CMR assessment of right ventricular function in pulmonary hypertension, including RV-FWLS measurement, CMR-derived RVEF calculation, pooled correlations, and factors affecting measurement reproducibility.

Figure 6 integrates the principal components of RV functional assessment used in this review, including the RV-FWLS measurement approach, calculation of CMR-derived RVEF, and the pooled correlations. RV-FWLS had the largest numerical pooled correlation (r = 0.79, 95% CI: 0.73–0.83), followed by RV-GLS (r = 0.75, 95% CI: 0.60–0.85) and RV-FAC (r = 0.72, 95% CI: 0.61–0.80). These values are presented descriptively because the analyses did not include a formal statistical comparison between the echocardiographic parameters. The illustration also identifies technical considerations that may influence measurement consistency, emphasizing standardized image acquisition, careful analysis, and appropriate operator expertise.

Figure 6 provides an integrated illustration of RV functional assessment using echocardiography and CMR. It depicts the RV-FWLS measurement workflow, the calculation of CMR-derived RVEF, and the pooled correlations evaluated in this meta-analysis. RV-FWLS demonstrated the numerically highest pooled correlation with CMR-derived RVEF (r = 0.79, 95% CI: 0.73–0.83), followed by RV-GLS (r = 0.75, 95% CI: 0.60–0.85) and RV-FAC (r = 0.72, 95% CI: 0.61–0.80). These differences are presented descriptively because no formal statistical comparison between parameters was performed. The figure also illustrates technical factors that may affect measurement consistency and emphasizes the importance of standardized image acquisition, careful interpretation, and appropriate expertise to improve reproducibility.

## 4. Discussion

This systematic review and meta-analysis examined how selected echocardiographic measures of RV function relate to CMR-derived RVEF in patients with PH. Across the available evidence, RV-FAC, RV-GLS, and RV-FWLS each showed significant positive associations with the CMR reference measure. These findings suggest that echocardiographic indices may provide clinically useful information about RV systolic performance in PH.

RV-GLS offers an objective assessment of RV systolic function and has been associated with prognosis, potentially providing information beyond conventional measures [14]. Two-dimensional speckle-tracking echocardiography is an established approach for measuring RV-GLS and has demonstrated feasibility and reproducibility in clinical practice [15]. Because strain analysis can identify relatively subtle myocardial dysfunction, RV-GLS may be useful for longitudinal surveillance. Assessment from multiple apical views may also improve characterization of RV systolic performance compared with conventional indices that predominantly reflect longitudinal shortening [9].

Our findings also support RV-FAC as a practical echocardiographic measure that is associated with CMR-derived RVEF. Previous investigations comparing echocardiographic and CMR measures of RV function have similarly reported moderate-to-strong relationships for RV-FAC [16,17,18,19]. RV-FAC has also demonstrated good reproducibility and may be less dependent on observer experience than some alternative measures, such as RV fractional shortening [19]. Three-dimensional echocardiographic assessment of RVEF has shown potential as well, although underestimation of true RV volumes has been reported [18].

RV-FWLS showed the strongest numerical pooled association with CMR-derived RVEF and, importantly, no detectable statistical heterogeneity among the three contributing studies. This consistency makes RV-FWLS a promising echocardiographic correlate of CMR-derived RVEF. Prior studies have likewise suggested that RV-FWLS may correlate more closely with RVEF than some conventional echocardiographic measures [17]. Other work has supported its role in RV assessment and reported stronger relationships with CMR-derived RVEF than those for selected conventional indices [15,20]. Nevertheless, only three studies informed this analysis, so the absence of heterogeneity should be interpreted cautiously.

The heterogeneity observed across RV functional measures may reflect differences in participant characteristics, PH etiology [21], and imaging methodology, including the use of two- versus three-dimensional echocardiography, speckle tracking, and different RV imaging views [22,23]. The included cohorts were clinically diverse, encompassing idiopathic PAH, chronic thromboembolic PH, congenital heart disease-associated PH, and other causes. These conditions may differ in RV loading, remodeling, disease severity, and treatment response, potentially affecting both echocardiographic indices and CMR-derived RVEF. Such clinical variation may partly explain the heterogeneity observed for RV-FAC and RV-GLS. Because relatively few studies were available and PH subtypes were unevenly represented, meaningful etiology-specific subgroup analyses were not feasible.

Methodological differences may have contributed further variability, including analysis software, image quality, strain-sign conventions, the interval between echocardiography and CMR, and inclusion of healthy controls in some cohorts. Previous work has also suggested that combining multiple echocardiographic indices, including measures such as RV outflow tract systolic excursion, may improve assessment of RV function and clinical risk [24,25]. As shown in Figure 6, standardized RV-focused acquisition, consistent contouring, clearly defined regions of interest, and appropriate analysis software may improve reproducibility and reduce measurement variation. These factors support the use of experienced or specialized centers when detailed echocardiographic and CMR assessment is required.

The observed correlations reinforce the role of echocardiography as an accessible method for evaluating RV function in PH. However, a correlation between two measurements indicates association rather than agreement and does not demonstrate that the modalities are interchangeable. RV-FWLS and the other echocardiographic indices should therefore be regarded as complementary measures rather than replacements for CMR-derived RVEF. CMR remains the reference modality because of its reproducibility and ability to provide volumetric and functional information [26,27]. Its cost, limited availability, examination duration, and patient-specific contraindications nonetheless make echocardiography particularly useful for initial and serial assessment in routine practice [28,29,30,31].

Clinically, the results emphasize the importance of ongoing RV functional assessment in PH, given the prognostic importance of RV dysfunction [32,33]. RV-FAC, RV-FWLS, and RV-GLS may be useful for serial monitoring when repeated CMR is unavailable or impractical. Their role in directing treatment, however, requires prospective validation. Future investigations should harmonize echocardiographic acquisition and analysis and establish clinically meaningful thresholds for RV-FAC, RV-FWLS, and RV-GLS across different PH populations [34,35,36].

Although RV-FWLS had the highest numerical pooled correlation with CMR-derived RVEF, this finding should not be interpreted as proof of superiority. Only a small number of studies were available; the cumulative sample was limited, and heterogeneity was moderate to substantial for RV-FAC and RV-GLS. These factors reduce confidence in the precision and generalizability of the pooled estimates. Moreover, correlation does not establish agreement or interchangeability, so RV-FWLS cannot currently be considered a definitive substitute for CMR-derived RVEF. Its findings instead support consideration of RV-FWLS as a practical adjunct when CMR cannot be performed. Larger prospective studies using standardized protocols are needed to confirm these associations and establish clinically useful thresholds.

Several limitations should be considered. First, heterogeneity varied by parameter, being absent for RV-FWLS but moderate or substantial for RV-FAC and RV-GLS. Differences in study design, participant characteristics, PH subtype, imaging acquisition, and strain-analysis software may have contributed. Variation in the reporting of signed strain values versus absolute strain magnitude could also influence correlation direction and comparability. The small number of contributing studies prevented reliable subgroup analyses and meta-regression that might otherwise clarify residual heterogeneity. The interval between echocardiography and CMR also varied and may have introduced additional measurement variability because RV loading and function can change over time. Most studies, however, performed the two examinations within a relatively short interval, which may have limited this effect.

This review has several strengths. We used a broad search across the databases specified in the Methods section, supporting comprehensive identification of potentially relevant studies. The review process followed PRISMA and Cochrane recommendations, with predefined eligibility criteria and structured data extraction. Heterogeneity was quantified using I^2^, and random-effects estimates were emphasized because clinical and methodological differences were expected. Evaluating RV-FAC, RV-FWLS, and RV-GLS within a common analytic framework allowed descriptive comparison with CMR-derived RVEF as the reference measure. Because no formal statistical test comparing the three echocardiographic parameters was performed, however, the apparent differences should be regarded as descriptive rather than evidence of superiority.

## 5. Conclusions

In patients with pulmonary hypertension, RV-FAC, RV-FWLS, and RV-GLS each demonstrated positive correlations with CMR-derived RVEF. RV-FWLS showed the largest numerical pooled correlation and the most consistent estimates, whereas RV-FAC and RV-GLS exhibited moderate-to-substantial heterogeneity. The absence of a formal statistical comparison means that the numerical advantage of RV-FWLS cannot be interpreted as demonstrated superiority over RV-GLS or RV-FAC. These findings indicate that all three echocardiographic measures may provide useful, accessible information about RV function, particularly when CMR is unavailable. CMR remains the reference method when accurate quantification of RV volume and RVEF is required. Further large, prospective studies using standardized imaging protocols are needed to confirm these relationships and improve clinical application of echocardiographic RV measures.

## Figures and Tables

**Table 1:** Baseline and Study Characteristics.

Author	Country	Study Design	Intervention Group	Control Group	Total Patients	Patients in the Intervention Group	Patients in the Control Group	Mean Age	Sex		Comorbidities
									Male *n*/%	Female *n*/%	(*n* = No. of Patients)
Evaldsson et al., 2019 [9]	Sweden	Comparative study	CMR	Echocardiography	90	55	35	62 ± 15	18	37	Idiopathic PAH (40 pts), Left Heart Disease (5), Chronic thromboembolic disease/other comorbid diseases (9 pts)
Yuman Li et al. 2020 [10]	China	prospective observational study.	Vasodilator therapies, including intravenous/inhaled prostacyclin analogs, phosphodiesterase-5 inhibitors, or endothelin receptor antagonists, or their combination, according to current guidelines.	Healthy volunteers	89	54	35	52 ± 12 (control group). 54 ± 16 (PAH).	-	-	congenital heart disease (5 pts), Idiopathic PAH (19 pts)
Li YD et al. 2015 [5]	China	retrospective correlational study.	Echocardiography	CMR	32	-	single-arm observational study of 32 CTEPH pts	54.50 ± 11.57 years	17	15	Chronic thromboembolic disease/other comorbid diseases (all participants)
Shiran et al. 2014 [11]	USA	Retrospective observational	Magnetic resonance imaging (MRI) and TTE images were retrospectively reviewed in 40 consecutive patients with pulmonary hypertension.	not mentioned	40	-	single-arm study of 40 pts with PH.	42 ± 12 years	6	34	congenital heart disease (5 pts), Idiopathic PAH (18 pts), Chronic thromboembolic disease/other comorbid diseases (3 pts), connective tissue disease (8 pts)
Yang et al. 2013 [12]	China	Prospective observational study	Idiopathic PAH, PAH associated with connective tissue diseases, hereditary PAH	not mentioned	30	idiopathic PAH 24 patients (80%); PAH associated with connective tissue diseases 5 patients (16.7%); hereditary PAH: 1 patient (3.3%)	N/A	30 ± 10	6 (20%)	24 (80%)	Idiopathic PAH (24 pts), Chronic thromboembolic disease/other comorbid diseases (1 pt), connective tissue disease (5 pts)
Liu et al. 2016 [13]	China	Observational study	mild pulmonary hypertension (PH) and moderate/severe PH; 2D speckle-tracking echocardiography and cardiac MR	Healthy volunteers	121	80	41	42.07 ± 7.51 years	37	43	N/A

**Table 2:** Summary of pooled results.

Outcome	Evidence Base	Random-Effect Estimate (95% CI)	Common-Effects Estimate (95% CI)	I^2^ (%)	τ^2^	*p* for Heterogeneity
Right Ventricular Fractional Area Change (RV-FAC) Correlation	5 studies; 331 participants	r = 0.72 (0.61–0.80)	r = 0.73 (0.68–0.78)	61.8	0.0298	0.0331
Right Ventricular Free-Wall Longitudinal Strain (RV-FWLS) Correlation	3 studies; 259 participants	r = 0.79 (0.73–0.83)	r = 0.79 (0.73–0.83)	0.0	0	0.9809
Right Ventricular Global Longitudinal Strain (RV-GLS) Correlation	3 estimates from 2 studies	r = 0.75 (0.60–0.85)	r = 0.75 (0.69–0.80)	80.3	0.0490	0.0062
Pooled Mean Right Ventricular Fractional Area Change (RV-FAC)	8 estimates from 5 studies; 376 observations	31.10% (26.77–35.43)	30.54% (29.58–31.49)	90.9	36.3947	<0.0001

## Data Availability

All data generated or analyzed during this study are included in the manuscript and its Supplementary File. All data analyzed during this study are derived from publicly available primary literature and available from the authors upon reasonable request.

## Supplementary Materials

The following supporting information can be downloaded at: https://www.mdpi.com/article/10.3390/diagnostics16182899/s1; Table S1: Baseline Cardiac MRI Parameters; Table S2: Baseline Right Ventricular Free Wall Longitudinal Strain of included studies; Table S3: Baseline Echocardiographic Parameters; Table S4: Quality assessment using the Newcastle-Ottawa Scale; Table S5. Reported Time Interval Between Echocardiographic and Cardiac Magnetic Resonance Assessments in the Included Studies; Table S6: PRISMA-2020 Checklist (refer to Figure 1 in manuscript for PRISMA Flow Diagram).

## Abbreviations

PH	Pulmonary hypertension
RV-FWLS	Right ventricular free wall longitudinal strain
RV-GLS	Right ventricular global longitudinal strain
CMR	Cardiac magnetic resonance
RVEF	Right ventricular ejection fraction
RV-FAC	Right ventricular fractional area change
I^2^	Higgins heterogeneity statistic (I-squared)

## References

[1] Schmeißer A., Rauwolf T., Groscheck T., Fischbach K., Kropf S., Luani B., Tanev I., Hansen M., Meißler S., Schäfer K. (2021). Predictors and prognosis of right ventricular function in pulmonary hypertension due to heart failure with reduced ejection fraction. ESC Heart Fail..

[2] Yogeswaran A., Rako Z., Yildiz S., Ghofrani H.A., Seeger W., da Rocha B.B., Gall H., Kremer N.C., Douschan P., Papa S. (2023). Echocardiographic evaluation of right ventricular diastolic function in pulmonary hypertension. ERJ Open Res..

[3] Labrada L., Vaidy A., Vaidya A. (2023). Right ventricular assessment in pulmonary hypertension. Curr. Opin. Pulm. Med..

[4] Li Q., Zhang Y., Cui X., Lu W., Ji Q., Zhang M. (2024). Optimal combination of right ventricular functional parameters using echocardiography in pulmonary arterial hypertension. ESC Heart Fail..

[5] Li Y.-D., Wang Y.-D., Zhai Z.-G., Guo X.-J., Wu Y.-F., Yang Y.-H., Lu X.-Z. (2015). Relationship between echocardiographic and cardiac magnetic resonance imaging-derived measures of right ventricular function in patients with chronic thromboembolic pulmonary hypertension. Thromb. Res..

[6] Hameed A., Condliffe R., Swift A.J., Alabed S., Kiely D.G., Charalampopoulos A. (2023). Assessment of right ventricular function—A state of the art. Curr. Heart Fail. Rep..

[7] Negru A., Tarcau B.M., Agoston-Coldea L. (2024). Cardiac magnetic resonance imaging in the evaluation of functional impairments in the right heart. Diagnostics.

[8] Page M.J., McKenzie J.E., Bossuyt P.M., Boutron I., Hoffmann T.C., Mulrow C.D., Shamseer L., Tetzlaff J.M., Akl E.A., Brennan S.E. (2021). The PRISMA 2020 statement: An updated guideline for reporting systematic reviews. BMJ.

[9] Evaldsson A.W., Ingvarsson A., Smith J.G., Rådegran G., Roijer A., Waktare J., Ostenfeld E., Meurling C. (2019). Echocardiographic right ventricular strain from multiple apical views is superior for assessment of right ventricular systolic function. Clin. Physiol. Funct. Imaging.

[10] Li Y., Wang T., Haines P., Li M., Wu W., Liu M., Chen Y., Jin Q., Xie Y., Wang J. (2020). Prognostic value of right ventricular two-dimensional and three-dimensional speckle-tracking strain in pulmonary arterial hypertension: Superiority of longitudinal strain over circumferential and radial strain. J. Am. Soc. Echocardiogr..

[11] Shiran H., Zamanian R.T., McConnell M.V., Liang D.H., Dash R., Heidary S., Sudini N.L., Wu J.C., Haddad F., Yang P.C. (2014). Relationship between echocardiographic and magnetic resonance derived measures of right ventricular size and function in patients with pulmonary hypertension. J. Am. Soc. Echocardiogr..

[12] Yang T., Liang Y., Zhang Y., Gu Q., Chen G., Ni X.-H., Lv X.-Z., Liu Z.-H., Xiong C.-M., He J.-G. (2013). Echocardiographic parameters in patients with pulmonary arterial hypertension: Correlations with right ventricular ejection fraction derived from cardiac magnetic resonance and hemodynamics. PLoS ONE.

[13] Liu Y., Wang D., Du Q., Che G., Tian J., Su Y. (2016). Evaluation of right ventricular systolic function in patients with chronic pulmonary heart disease by 2-dimensional speckle-tracking echocardiography. J. Ultrasound Med..

[14] Lee J.-H., Park J.-H. (2018). Strain analysis of the right ventricle using two-dimensional echocardiography. J. Cardiovasc. Imaging.

[15] Muraru D., Haugaa K., Donal E., Stankovic I., Voigt J.U., Petersen S.E., Popescu B.A., Marwick T. (2022). Right ventricular longitudinal strain in the clinical routine: A state-of-the-art review. Eur. Heart J. Cardiovasc. Imaging.

[16] Anavekar N.S., Gerson D., Skali H., Kwong R.Y., Yucel E.K., Solomon S.D. (2007). Two-dimensional assessment of right ventricular function: An echocardiographic-MRI correlative study. Echocardiography.

[17] Focardi M., Cameli M., Carbone S.F., Massoni A., De Vito R., Lisi M., Mondillo S. (2015). Traditional and innovative echocardiographic parameters for the analysis of right ventricular performance in comparison with cardiac magnetic resonance. Eur. Heart J. Cardiovasc. Imaging.

[18] Hamilton-Craig C.R., Stedman K., Maxwell R., Anderson B., Stanton T., Chan J., Yamada A., Scalia G.M., Burstow D.J. (2016). Accuracy of quantitative echocardiographic measures of right ventricular function as compared to cardiovascular magnetic resonance. Int. J. Cardiol. Heart Vasc..

[19] Caudron J., Fares J., Vivier P.-H., Lefebvre V., Petitjean C., Dacher J.-N. (2011). Diagnostic accuracy and variability of three semi-quantitative methods for assessing right ventricular systolic function from cardiac MRI in patients with acquired heart disease. Eur. Radiol..

[20] Smolarek D., Gruchala M., Sobiczewski W. (2017). Echocardiographic evaluation of right ventricular systolic function: The traditional and innovative approach. Cardiol. J..

[21] Fontes Oliveira M., Trepa M., Costa R., Frias A.D., Silveira I., Cabral S., Santos M., Torres S., Reis A. (2020). P1505 Echocardiographic assessment of different pulmonary hypertension groups. Eur. Heart J. Cardiovasc. Imaging.

[22] Vitarelli A., Mangieri E., Terzano C., Gaudio C., Salsano F., Rosato E., Capotosto L., D’ORazio S., Azzano A., Truscelli G. (2015). Three-dimensional echocardiography and 2D-3D speckle-tracking imaging in chronic pulmonary hypertension: Diagnostic accuracy in detecting hemodynamic signs of right ventricular (RV) failure. J. Am. Heart Assoc..

[23] Rajagopal S., Forsha D.E., Risum N., Hornik C.P., Poms A.D., Fortin T.A., Tapson V.F., Velazquez E.J., Kisslo J., Samad Z. (2014). Comprehensive assessment of right ventricular function in patients with pulmonary hypertension with global longitudinal peak systolic strain derived from multiple right ventricular views. J. Am. Soc. Echocardiogr..

[24] Hwang J.-W. (2019). Assessment of right ventricular systolic function: Conventional methods and modified tricuspid annular plane systolic excursion. J. Cardiovasc. Imaging.

[25] Lo Giudice F., Escribano-Subias P., Tello K., Kopec G., Ghio S., Giannakoulas G., D’aLto M., Filomena D., Manzi G., Orlando A. (2025). Echocardiography of the right heart in pulmonary arterial hypertension: Insights from the ULTRA RIGHT VALUE study. Eur. Heart J. Imaging Methods Pract..

[26] Baur L.H.B. (2008). Magnetic resonance imaging: The preferred imaging method for evaluation of the right ventricle. Int. J. Cardiovasc. Imaging.

[27] Tadic M. (2015). Multimodality evaluation of the right ventricle: An updated review. Clin. Cardiol..

[28] Couto M., Souto M., Martínez A., Martinez A., Vieira C., Pumar J.M., Croisille P. (2020). Accuracy of right ventricular volume and function assessed with cardiovascular magnetic resonance: Comparison with echocardiographic parameters. Clin. Imaging.

[29] Li Y., Zhang L., Gao Y., Wan X., Xiao Q., Zhang Y., Sun W., Xie Y., Zeng Q., Chen Y. (2021). Comprehensive assessment of right ventricular function by three-dimensional speckle-tracking echocardiography: Comparisons with cardiac magnetic resonance imaging. J. Am. Soc. Echocardiogr..

[30] Tsipis A., Petropoulou E. (2022). Echocardiography in the evaluation of the right heart. US Cardiol. Rev..

[31] Kim B.-J., Thomas J.D. (2025). Echocardiographic parameters of the right ventricle in patients with pulmonary hypertension: A review. Korean Circ. J..

[32] Murata M. (2021). Clinical significance of right ventricular function in pulmonary hypertension. Keio J. Med..

[33] Badagliacca R., Papa S., Poscia R., Pezzuto B., Manzi G., Torre R., Fedele F., Vizza C.D. (2018). The importance of right ventricular function in patients with pulmonary arterial hypertension. Expert Rev. Respir. Med..

[34] Forfia P., Benza R., D’Alto M., De Marco T., Elwing J.M., Frantz R., Haddad F., Oudiz R., Preston I.R., Rosenkranz S. (2023). The heart of the matter: Right heart imaging indicators for treatment escalation in pulmonary arterial hypertension. Pulm. Circ..

[35] Mukherjee M., Rudski L.G., Addetia K., Afilalo J., D’ALto M., Freed B.H., Friend L.B., Gargani L., Grapsa J., Hassoun P.M. (2025). Guidelines for the echocardiographic assessment of the right heart in adults and special considerations in pulmonary hypertension: Recommendations from the American Society of Echocardiography. J. Am. Soc. Echocardiogr..

[36] Venkatachalam S., Wu G., Ahmad M. (2017). Echocardiographic assessment of the right ventricle in the current era: Application in clinical practice. Echocardiography.

